# Analysis of strain relatedness using High Resolution Melting in a case of recurrent candiduria

**DOI:** 10.1186/1471-2180-13-13

**Published:** 2013-01-23

**Authors:** Sara Gago, Belen Lorenzo, Alicia Gomez-Lopez, Isabel Cuesta, Manuel Cuenca-Estrella, Maria J Buitrago

**Affiliations:** 1 Servicio de Micología, Centro Nacional de Microbiología, Instituto de Salud Carlos III, Ctra Majadahonda-Pozuelo, Km 2, Majadahonda, Madrid, 28220, Spain; 2 Servicio de Microbiología, Hospital Virgen de la Concha, Zamora, España; 3 Unidad de Bioinformática, Centro Nacional de Microbiología, Instituto de Salud Carlos III, Madrid, Spain

**Keywords:** Candiduria, High resolution melting, Genotyping

## Abstract

**Background:**

Several genotyping protocols have been described to study *Candida albicans* strains with different sensitivity values. In this study we have analyzed the genetic relatedness and the antifungal susceptibility of several *Candida albicans* strains isolated from a patient who from suffered recurrent candiduria for a period of five years. Strains were genotyped using Microsatellite Length Polymorphism (MLP) with three microsatellite markers (HIS 3, EF 3 and CDC 3), and a new method based on high resolution melting (HRM) was developed to analyze the microsatellite region. This method was compared with the conventional technique that uses capillary electrophoresis.

**Results:**

MICs of the isolates showed the existence of fluconazole susceptible and resistant strains. An inter-colony test using single concentration (8 and 16 mg/l) of fluconazole revealed the coexistence of both fluconazole susceptible and resistant strains. Both genotyping analysis methods showed that all the patient’s isolates had a clonal origin. HRM analysis method developed was able to accurately establish strain relatedness and presented a discriminatory power of 0.77.

**Conclusions:**

Although HRM analysis method presented a lower discriminatory power compared to methods based on capillary electrophoresis, it provided a more cost-effective and suitable alternative for genotyping *C. albicans* in a clinical laboratory.

## Background

*Candida albicans* is a ubiquitous commensal in healthy individuals; it is, however, a very important opportunistic pathogen for immunologically weak and immuno-compromised people [1]. Recurrent and/or persistent infections by *Candida* species are frequent, particularly in oropharyngeal and vaginal candidiasis, although it has also been described in urinary tract infections [2]. Studies describing recurrent infections have focused on determining the relatedness between colonizing and infective strains [3,4], as well as between successive infective strains [5-9]. It seems clear now that the majority of commensal and infecting populations of *C*. *albicans* from the same individuals are clonal in origin but subsequently undergo microevolution at the site of colonization and through recurrent episodes of infection [5,10,11]. The microevolution of the strains is a frequent process in recurrent infections and it takes place in response to adaptive changes [9,12]. A recent work which examined the “in vitro” dynamics of *C. albicans* populations in the presence or absence of fluconazole has shown that mutations that lead to increased drug resistance appear frequently [13]. Others authors suggest that natural *C. albicans* populations comprise a mixture of closely related strain types [6].

Typing methods have been described as useful tools for the differentiation between strains isolated only once and those able to cause recurrent infections. Although several typing methods have been described for *C. albicans* (AFLP, RFLP-PCR or MLST), one of the most suitable is the fragment length analysis of microsatellites called Microsatellite Length Polymorphism (MLP). This technique has a high discriminatory power and reproducibility. MLP analysis has proved its efficacy and reproducibility in a large number of epidemiological studies [9,14-19]; however, this technique is not easy to use and the estimated cost per isolate remains high.

The High Resolution Melting (HRM) provides a faster and cheaper method for microsatellite fragment analysis. This technique uses fluorescent DNA binding dyes with improved saturation properties allowing a precise assessment of sequence variation based on DNA melting curves analysis [20,21]. The suitability of HRM to discriminate PCR products based on one nucleotide change has also been described. Some recent articles, focusing on the capacity of HRM to identify and genotype fungi, have been reported [15,22].

In this work, we developed a method based on HRM to assess the relatedness of strains in a clinical case of recurrent candiduria. The results were compared with the conventional MLP genotyping techniques. The isolates, recovered over a period of five years, additionally showed significant differences in their susceptibility to antifungal agents. Antifungal susceptibility test and selection of resistant population was performed.

## Methods

### Origin of the strains and clinical data from the patient

The strains were isolated from a 62 year old male with medullary sponge right kidney (Carchi-Ricci disease) and recurrent reno-urethral lithiasis subjected to several lithotripsies. The patient was admitted in a Tertiary General Hospital (Hospital Virgen de la Concha, Zamora, Spain) diagnosed with right pyelonephritis caused by obstructive kidney stones. *C. albicans* was isolated in blood cultures and urocultures. The antifungal susceptibility profile showed that azoles and amphotericin B were active in vitro against this strain (CNM-CL-4929, Yeast Collection of the Spanish National Centre for Microbiology) as shown in Table 1.

**Table 1:** MICs values and isolation data of the clinical isolates used in this study

**Strain**	**Isolate origin**	**Isolation data**	**AB**	**5FZ**	**FZ**	**IZ**	**VZ**	**PZ**	**CA**	**MC**	**AN**	
CNM-CL-4929	Blood culture	02-28-2003	0.03	0.12	0.12	0.015	0.015	*	*	*	*	
L06/31	Urine	02-01-2006	0.12	0.12	0.5	0.015	0.015	0.015	0.03	0.03	0.03	
L06/32	Urine	02-01-2006	0.12	0.12	0.5	0.015	0.015	0.015	0.03	0.03	0.03	
CNM-CL-6188^#^	Urine	08-18-2006	0.25	0.12	> 64	> 8	> 8	> 8	0.25	0.03	0.03	
L06/260	Urine	08-18-2006	0.06	0.12	8	1	0.12	1	0.03	0.03	0.03	
L06/349	Urine	11-07-2006	0.06	0.12	0.25	0.015	0.015	0.015	0.12	0.03	0.03	
L06/350	Urine	11-07-2006	0.06	0.12	0.25	0.015	0.015	0.015	0.12	0.03	0.03	
CNM-CL-6361^#^	Urine	03-20-2006	0.25	0.12	> 64	> 8	> 8	> 8	0.25	0.03	0.03	#
CNM-CL-6373^#^	Urine	04-16-2006	0.12	0.12	> 64	1	> 8	4	0.25	0.03	0.03	
L07/130	Urine	04-16-2006	0.12	128	16	16	16	16	0.25	0.03	0.03	
CNM-CL-6399^#^	Urine	05-21-2007	0.25	0.12	> 64	> 8	> 8	> 8	0.25	0.03	0.03	#
CNM-CL-6431^#^	Urine	06-13-2007	0.25	0.12	> 64	> 8	> 8	> 8	0.25	0.03	0.03	#
CNM-CL-6488^#^	Urine	07-27-2007	0.12	0.12	0.25	0.015	0.015	0.015	0.25	0.03	0.03	#
L07/453	Urine	11-21-2007	0.03	0.12	0.12	0.015	0.015	0.015	0.12	0.03	0.03	
L07/454	Urine	11-21-2007	0.03	0.12	0.25	0.06	0.06	0.12	0.06	0.03	0.03	
CNM-CL-6714^#^	Urine	03-07-2008	0.25	0.12	> 64	0.06	> 8	> 8	0.25	0.03	0.03	#
CNM-CL-7019^#^	Urine	11-12-2008	0.12	0.12	2	0.12	0.015	0.06	0.25	0.03	0.03	#
CNM-CL-7020^#^	Urine	11-12-2008	0.25	0.12	0.25	0.015	0.015	0.015	0.25	0.03	0.03	#

# Strains included in the genotyping analysis.

* No MICs data available.

CNM-CL Yeast Collection of the Spanish National Center for Microbiology.

Treatment with ciprofloxacin 400 mg/12 h and fluconazole iv 200 mg/12 h was started. After three days of treatment, as fever persisted and blood and urine cultures remained positive, fluconazole was replaced by amphotericin B lipid complex 200 mg/24 h iv and 100 mg every other day. Six days after admission, lithotripsy was performed and a double J stent was placed. He was discharged from hospital a month after admission. From 2003 to 2008, the patient suffered from several episodes of *Candida* infection and underwent multiple lithotripsies. He was treated with oral fluconazole (200 mg/12 h) several times. A total of 18 strains were isolated from urine and sent to the Mycology Reference Laboratory for identification and susceptibility testing (Table 1). In 2006 the susceptibility of one of the isolates (CNM-CL-6188) showed high MICs for fluconazole (>8 mg/l). Three months later, the patient was readmitted to the hospital and diagnosed with hydronephrosis and kidney obstruction after the placement of a double J stent in the right kidney. The stent was removed and a percutaneous nephrostomy was performed. An initial treatment with caspofungin 70 mg iv and then 50 mg iv/24 h was established. Later, the patient was treated with amphotericin B lipid complex (11 days) and was subsequently put back on caspofungin until patient was discharged from the hospital. During the stay in the hospital, blood cultures were negative while urine cultures remained positive until the patient was treated with amphotericin B. The patient’s isolates were controlled in an outpatient mode up to the end of 2008, at which time the patient went to another institution and no more samples were taken**.** The written informed consent was sought and obtained from the patient according to Spanish regulations at that date. The patient also signed his consent to the release of his clinical and personal information in a scientific publication.

### Antifungal susceptibility testing

Antifungal susceptibilities were tested in vitro according to the EUCAST microdilution method (AFST-EUCAST, definitive document 7.1). Interpretative breakpoints proposed by EUCAST for fluconazole and voriconazole were used [23]. For the rest of the antifungal tested, the breakpoints proposed by Rodriguez-Tudela et al. were used [24]. The antifungal agents used were amphotericin B, flucytosine, fluconazole, itraconazole, voriconazole, posaconazole, caspofungin, micafungin, and anidulafungin. Isolates were stored at −20°C until use.

### Selection of resistant population

In February of 2011, the isolates available in our culture collection (Tables 1 and 2) were subcultured for genotyping studies. To analyze the probability of the coexistence of fluconazole resistant and susceptible populations in each isolate, we performed a screening assay based on a single-concentration fluconazole test [25]. The antifungal concentration used in this assay was selected on the basis of the MIC values previously obtained. The test of growth was performed in microplates containing RPMI 1640 medium supplemented with 2% glucose (Sigma-Aldrich, Madrid, Spain) and a final fluconazole concentration of 8 and 16 mg/l. Ten colonies of each isolate were tested. For each colony, a suspension of 10^5^ cfu/ml was prepared. Plates were inoculated with 0.1 ml from the cell suspension. A growth control was also included. The Optical Density (OD) at 530 nm was measured after 24 and 48 hours of incubation. The reduction of the OD below 50% compared to control was considered as susceptibility to fluconazole.

**Table 2:** Intercolony fluconazole susceptibility in single concentration microdilution plates

	**No of colonies fluconazole resistant**	
**Strain**	**8 mg /l**	**16 mg/l**
CNM-CL-6188	2/10	1/10
CNM-CL-6361	5/10	4/10
CNM-CL-6373	9/10	9/10
CNM-CL-6399	10/10	4/10
CNM-CL-6431	2/10	2/10
CNM-CL-6488	0/10	0/10
CNM-CL-6714	4/10	4/10
CNM-CL-7019	0/10	0/10
CNM-CL-7020	0/10	0/10

CNM-CL Yeast Collection of the Spanish National Center for Microbiology.

### Genotyping studies

Nine representative strains isolated from the patient on different days were selected for performance of genotyping studies (Tables 1 and 3). The control population consisted of 20 strains from patients geographically and temporally unrelated. Nineteen out of 20 isolates were from whole blood and the remaining isolate was from pleural fluid (Table 3). ATCC64548 and ATCC64550 *C. albicans* reference strains were also included in this study. All isolates were identified by physiological and morphological tests, including microscopic examination and biochemical tests. The identification was confirmed by sequence analysis of the ITS (internal transcribed spacer) region of the rDNA [26].

**Table 3:** Microsatellite lenght (bp) for the three microsatellite markers using capillary electrophoresis

**Strain**	**Isolate origin**	**Length (bp) determined by PCR analysis of microsatellite markers:**		
		**CDC 3**	**EF 3**	**HIS 3**
CNM-CL-7426^a^	Whole blood	117/125	125/125	162/186
CNM-CL-7449^a^	Whole blood	117/125	125/125	162/190
CNM-CL-7470^a^	Whole blood	117/125	120/120	162/227
CNM-CL-7471^a^	Whole blood	117/117	130/130	162/162
CNM-CL-7478^a^	Whole blood	117/125	120/120	202/202
CNM-CL-7484^a^	Whole blood	125/125	125/125	162/190
CNM-CL-7498^a^	Whole blood	125/129	130/139	149/166
CNM-CL-7499^a^	Whole blood	117/129	130/139	154/154
CNM-CL-7503^a^	Whole blood	117/117	126/138	153/182
CNM-CL-7504^a^	Whole blood	117/117	124/130	149/166
CNM-CL-7513^a^	Whole blood	121/125	124/137	158/158
CNM-CL-7617^a^	Whole blood	117/117	124/130	313/313
CNM-CL-7624^a^	Whole blood	117/117	126/138	153/153
CNM-CL-7620^a^	Whole blood	117/125	120/120	162/210
CNM-CL-7640^a^	Whole blood	125/129	130/137	149/166
CNM-CL-7643^a^	Pleural fluid	117/117	124/130	149/166
CNM-CL-7683^a^	Whole blood	117/125	120/129	162/210
CNM-CL-7694^a^	Whole blood	117/129	130/139	148/153
CNM-CL-7705^a^	Whole blood	117/117	124/130	---/---
CNM-CL-7712^a^	Whole blood	117/125	120/129	162/210
ATCC64548^a^	Whole blood	113/113	124/124	162/162
ATCC64550^a^	Whole blood	117/125	120/129	162/178
CNM-CL-6188^b^	Urine	121/121	127/129	153/153
CNM-CL-6361^b^	Urine	121/121	127/129	153/153
CNM-CL-6373^b^	Urine	121/121	127/129	153/153
CNM-CL-6399^b^	Urine	121/121	127/129	153/153
CNM-CL-6431^b^	Urine	121/121	127/129	153/153
CNM-CL-6488^b^	Urine	121/121	127/129	153/153
CNM-CL-6714^b^	Urine	121/121	127/129	153/153
CNM-CL-7019^b^	Urine	121/121	127/129	153/153
CNM-CL-7020^b^	Urine	121/121	127/129	153/153

CNM-CL Yeast Collection of the Spanish National Center for Microbiology.

a: Control population.

b: strains from the case study included for genotyping studies.

Yeast cells were grown for 24 hours in Sabouraud broth medium at 30°C. Genomic DNA was extracted using a phenol:chloroform method [27] followed by purification using Chroma SPIN + TE 400 columns according to the manufacturer’s instructions (Clontech Laboratories, Becton Dickinson, Madrid, Spain).

Genotyping analysis of *C. albicans* was performed using MLP procedure with three different markers previously described, CDC 3 [28]; EF 3 [29] and HIS 3 [30]. The primers sequences used to amplify these markers were: CDC3 forward (5^′^ CAGATGATTTTTTGTATGAGAAGAA3^′^) and reverse (5^′^ CAGTCACAAGATTAAAATGTTCAAG3^′^); EF3 forward (5 TTTCCTCTTCCTTTCATATAGAA3^′^) and reverse (5 GGATTCACTAGCAGCAGACA3^′^) and HIS3 forward (5 TGGCAAAAATGATATTCCAA3^′^) and reverse (5^′^ TACACTATGCCCCAAACACA 3^′^).

MLP analysis using capillary electrophoresis was modified from Botterel et *al.*[14]. Alleles were amplified in a multiplex PCR in a 50 μl final volume containing 20 ng DNA, 1X PCR-Buffer II (Applied Biosystems, Madrid, Spain), 0.2 mM of each deoxynucleotide triphosphate, 5 mM of MgCl_2_, and 0.15 μM of each primer and 1U of AmpliTaq Polymerase (Applied Biosystems).

Sense CDC3 primer was labelled with 4, 7, 2^′^, 4^′^, 5^′^, 7^′^-hexachloro-6-carboxyfluorescein (HEX), EF3 antisense primer with 6-carboxyfluorescein (FAM) and HIS 3 sense primer was labelled with 2^′^-chloro-5^′^-fluoro-7^′^,8^′^-fused phenyl-1.4-dichloro-6-carboxyfluorescein (NED). Primers were synthesized by Sigma-Aldrich (Sigma-Aldrich, Madrid, Spain).

PCR reactions were performed in a GeneAmp PCR system 9700 (Applied Biosystems). The cycling conditions included a first step for preincubation (activation of the enzyme) and denaturation of the DNA template at 95°C during 5 minutes. Next steps consisted in an amplification program of 30 cycles as follow: denaturation at 95°C for 30 s, annealing at 55°C for 30 s and extension at 72°C for 1 min with a final extension step of 7 min at 72°C.

To assess the size of the fragments, 1 μl of the PCR products was added to 9 μl of Formamide Hi-Di (Applied Biosystems, Madrid, Spain) and 1 μl of the internal size standard ROX 500 (Applied Biosystems, Madrid, Spain). Capillary electrophoresis was run using the ABI 3730 XL (Applied Biosystems, Madrid, Spain) sequencer. Fragment size for the different alleles was calculated with GeneMapper version 3.0 (Applied Biosystems, Madrid, Spain).

**Figure 1:**
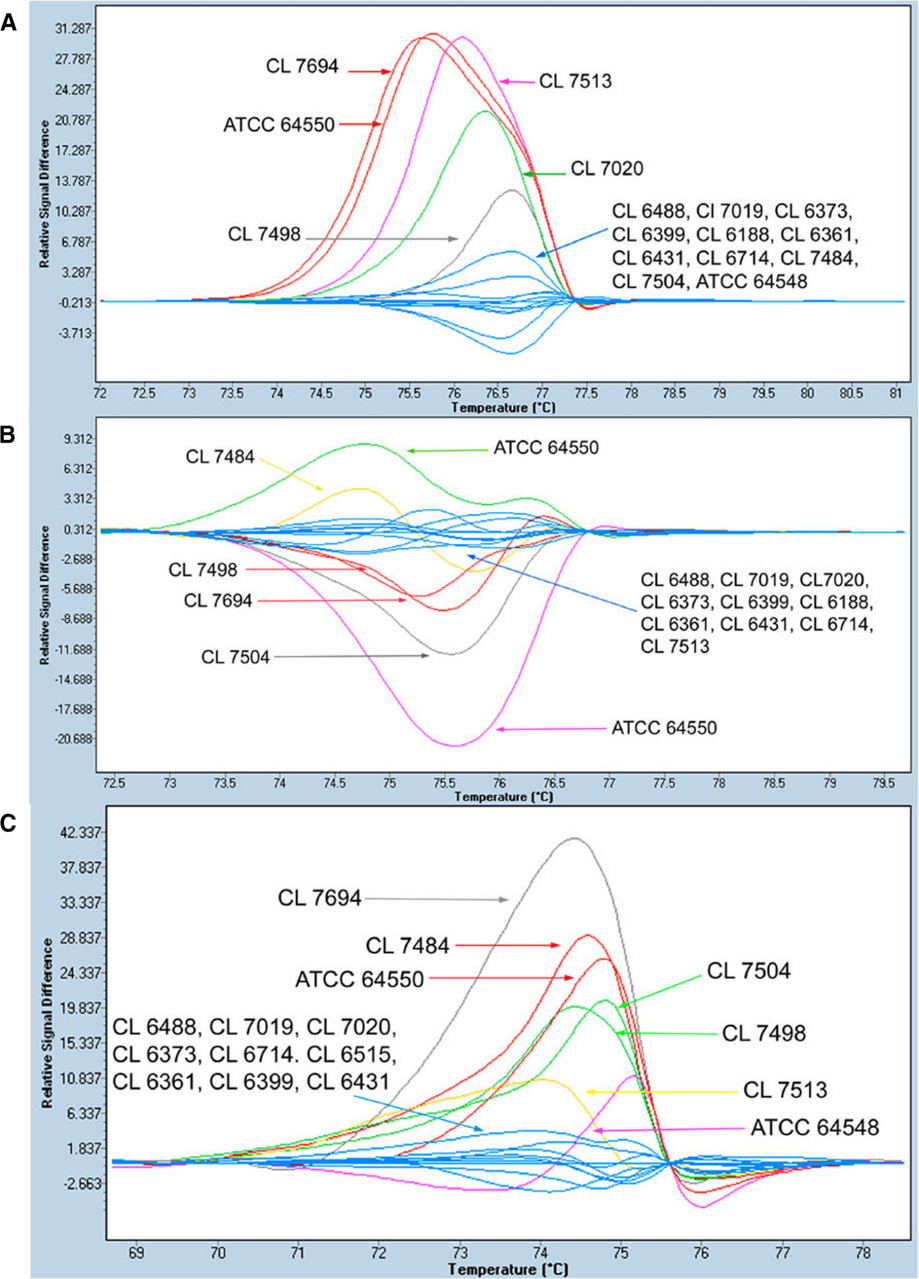
**Difference plots for the normalized and temperature shifted melting curves for microsatellite from control population and patient strains. A**) CDC3 marker; **B**) EF3 marker and **C**) HIS3 marker.

In addition, a HRM-based analysis was performed using singleplex PCRs with each pair of primers without any modification of the reaction conditions. Control population was selected based on MLP results. Strains included as control were: CL 7484, CL 7498, CL 7504, CL 7513, CL 7694, ATCC 64548 and ATCC 64550 (Figure 1). Seven different genotypes for the three markers were chosen (Figure 1). 

After PCR, HRM analysis was performed in a LightCycler 480 system (Roche, Madrid, Spain). To obtain the HRM curves, 1 μl of LightCycler® 480 ResoLight Dye (Roche, Madrid, Spain) was added to PCR products and the reactions were incubated at 95°C 1 min, followed by a renaturation step of 40°C for 1 min. Melting curves were generated by ramping from 65° to 95° at 0.02°C/s, 25 acquisitions/°C. HRM curves were plotted using the automated grouping option provided by the software and by manual editing for each microsatellite marker. Normalization conditions for each microsatellite marker are shown in Table 4.

**Table 4:** High resolution melting conditions and discrimination power

**HRM Marker**	**Pre-Melt T**	**Post-Melt T**	**Threshold/Sensitivity**	**DP**
CDC 3	71.95-72.96	79.34-81.11	5.645/0.3	0.53
EF 3	70.8-72.62	78.46-79.71	5.645/0.3	0.62
HIS 3	68.65-69.82	77.42-78.56	5.645/0.3	0.68
Multiplex				0.77

For sequencing, amplicons were treated with ExoSap –IT (GE Health Care, Madrid, Spain) following the manufacturer’s instructions. Sequencing reactions were performed in a GeneAmp PCR system 9700 (Applied Biosystems). Sequences were analyzed in triplicate.

The numerical index of discriminatory power for each marker and for the multiplex analysis was calculated in both genotyping analysis using the Simpson biodiversity index (D) [31]. The percentage of heterozygosis has been calculated by the ratio number of heterozygous genotypes/ total number of genotypes.

## Results

### Antifungal susceptibility testing

Antifungal susceptibility results are shown in Table 1. At first, isolates were susceptible to all antifungal agents tested; however, in August 2006 an isolate showed an azole-resistant phenotype and subsequently isolates susceptible and resistant to azoles appeared at random. Between March 2006 and June 2007 all strains tested were azole-resistant but this pattern changed again between July and November 2007. The latest azole resistant strain recovered was from March 2008.

### Fluconazole resistance selection

Ten colonies of each of the nine isolates genotyped were tested for fluconazole resistance at 8 and 16 mg/l final concentration. From five out of the 9 strains we were able to select resistant and susceptible isolates. On the other hand, from one strain all colonies were resistant and from the remaining three strains all checked colonies were susceptible to fluconazole in a final concentration of 8 mg/l. When fluconazole concentration was increased to 16 mg/l, the number of resistant colonies was reduced (Table 2).

### Genotyping studies

#### Microsatellite length genotyping

Microsatellite markers were used to genotype the nine strains recovered from the patient. Each PCR product was assigned to an allele [14] so each strain was characterized by 6 alleles that were differently coupled (Table 3). Strains from the patient showed the same microsatellite pattern for the three markers and they were different from the control population (Table 3). All the isolates recovered from the patient were homozygous for CDC 3 and HIS 3 markers while they showed a heterozygous genotype for EF 3 (Table 5).

**Table 5:** Characteristics of the microsatellite loci analyzed by capillary electrophoresis

**Microsatellite Marker**	**No. of alleles**	**No. of genotypes**	**No. of heterozygotic genotypes**	**DP**	**% heterozygosity**
CDC 3	5	8	4	0.81	50.00
EF 3	10	11	7	0.86	63.63
HIS 3	14	15	11	0.88	53.30
Multiplex				0.92	

The D value for EF3 was 0.86, similar to that previously reported [14,15], for CDC 3 it was 0.81, and for HIS 3 it was 0.87. The combination of three markers yielded a discriminatory power of 0.92 (Table 5).

#### Microsatellite HRM genotyping

When analysis by HRM was performed, isolates from the patient were grouped together for the three markers analyzed (Figure 1).

The analysis of marker CDC 3 showed that all homozygous strains, including those from the patient, were plotted in one group except for the CNM- CL 7020 strain (Figure 1A). Due to the unexpected result for CNM-CL7020, the PCR product was sequenced (6x sequence coverage) and a 3 bp insertion at 67 pb from the forward primer was found. Heterozygous strains were distributed in four groups according to their fragment length. The heterozygous strains CNM-CL 7694 and ATCC 64550 were plotted together although one of the alleles were different (Table 3). When we performed EF 3 fragments analysis by HRM, six different groups were plotted one of them contained strains from the patient while the control population was distributed into five groups according to its fragment size or whether they were homozygous or heterozygous (Figure 1B).

Finally, HRM analysis of the HIS3 marker showed six different groups. Strains from the patient were grouped together again. Strains in the control population were grouped based on their fragment size pattern (Figure 1C).

Discrimination power for CDC 3 marker was 0.53, for EF 3 it was 0.62 and for the HIS 3 marker it was 0.68. The combination of the three markers provided a DP value of 0.77 (Table 4).

## Discussion

Typing methods have been described as useful tools for the differentiation between strains isolated only once and those able to cause recurrent infections. Several methods have been developed to analyze microevolution and structure of *C. albicans* species. Although MLST (MultiLocus Sequence Typing) has been chosen as the most discriminatory technique [5,32], several articles have recently pointed towards the suitability of MLP [14-16,29]. In this study, nine isolates from a case of recurrent urinary infection were genotyped using microsatellites and a new HRM analysis method. Antifungal susceptibility testing revealed that strains from the patient were susceptible and resistant in vitro to fluconazole in a random way. Microvariation between colonies due to exposure of *C. albicans* to azole antifungal agents has been widely described [10,16] and the need to perform intercolony assays has also been reported [25,33,34]. We performed an inter-colony test modified from Schoofs et al. [25] and we were able to prove the coexistence of colonies resistant and susceptible to azoles in a high number of the strains tested. The number of azole-resistant colonies was variable depending on azole concentration.

A genotyping method based on HRM analysis was developed taking into account previous works showing that if the number of genotypes is higher than seven, the curve definition is not the best possible [35]. Based on that premise, for each marker we selected seven strains with different genotype, previously analysed by capillary electrophoresis. *C. albicans* microsatellites (CDC3, EF3 and HIS3) were amplified using LightCycler® 480 ResoLight as intercalating dye. When HRM analysis was performed, the isolates from the patient were plotted together and these results were consistent with those obtained by capillary electrophoresis. There was only one exception for the CDC3 marker where one strain (CNM-CL7020) was not grouped, as expected, with the other strains showing the same MLP genotype. The sequence of the fragment showed a 3 bp insertion that explained the melting differences. This fact supports previous works in which HRM allowed to identify changes in the sequence length and one nucleotide changes [36].

Although the calculated discrimination power was higher for the analysis using capillary electrophoresis than for HRM analysis (0.92 vs. 0.77) as previously reported [14]. The HRM analysis showed several advantages; it was a very simple and fast technique and results were obtained in 3 hours (including amplification), the interpretation of results was easy and the cost per sample was much lower than MLP genotyping due to this technique does not require sequencing equipment and the primers are not end-labelled. Our estimate is that the cost per sample using capillary electrophoresis is more than twice that of using HRM analysis. Furthermore, it can be used in a routine laboratory setting as it only requires real time PCR equipment. In this study, although we were not able to demonstrate the mechanism underlying the variability in the susceptibility to azoles in the strains tested, we were able to confirm that resistant and susceptible isolates were genetically closely related with an easy method to analyze microsatellites. The results highlight the need for more in-depth studies to be performed on these kinds of infections for an accurate and appropriate management thereof.

## Conclusions

This method is a useful tool for performing a fast screening to establish relatedness between strains in outbreaks or surveillance studies in cases of recurrent or persistent infections. To our knowledge, this is the first study in which three microsatellite markers were analyzed by HRM by using seven strains with different genotype as control population and reaching HRM resolution limits. Although HRM analysis method presented a lower degree of discrimination compared to other genotyping methods, it provided a more cost-effective and suitable alternative for genotyping *C. albicans* in a clinical laboratory.

## Competing interest

In the past 5 years, M.C.E. has received grant support from Astellas Pharma, bioMerieux, Gilead Sciences, Merck Sharp and Dohme, Pfizer, Schering Plough, Soria Melguizo SA, the European Union, the ALBAN program, the Spanish Agency for International Cooperation, the Spanish Ministry of Culture and Education, The Spanish Health Research Fund, The Instituto de Salud Carlos III, The Ramon Areces Foundation, The Mutua Madrileña Foundation. He has been an advisor/consultant to the Panamerican Health Organization, Gilead Sciences, Merck Sharp and Dohme, Pfizer, and Schering Plough. He has received remuneration for talks on behalf of Gilead Sciences, Merck Sharp and Dohme, Pfizer, and Schering Plough.

## Authors’ contributions

SG performed the genotyping studies, the analysis of the results and also participated in drafting the manuscript. BL participated in the collection of clinical data and strains from the patient. AG-L has been involved in the antifungal susceptibility testing. IC has made contributions to the analysis of the results. MC-E has been involved in drafting the manuscript and in the final approval of the version to be published following a critical review thereof. MJB was responsible for the original design of the study and participated in its further design and development as well as having been involved in drafting the manuscript. All authors have read and approved the final manuscript.
